# Clinical value of combined detection of procalcitonin, D-dimer and fibrinogen in the evaluation of children with sepsis

**DOI:** 10.12669/pjms.41.7.11195

**Published:** 2025-07

**Authors:** Ximeng Hao, Hongnian Duan, Lei Shi, Chao-nan Fan, Xin-Hui Liu, Jiang-tao Ma

**Affiliations:** 1 Ximeng Hao Baoding Pediatric Severe Infectious Diseases Research Laboratory, Baoding Hospital of Beijing Children’s Hospital, Capital Medical University, Baoding 071000, Hebei, China; 2 Hongnian Duan Baoding Pediatric Severe Infectious Diseases Research Laboratory, Baoding Hospital of Beijing Children’s Hospital, Capital Medical University, Baoding 071000, Hebei, China; 3 Lei Shi Baoding Pediatric Severe Infectious Diseases Research Laboratory, Baoding Hospital of Beijing Children’s Hospital, Capital Medical University, Baoding 071000, Hebei, China; 4 Chao-nan Fan Baoding Pediatric Severe Infectious Diseases Research Laboratory, Baoding Hospital of Beijing Children’s Hospital, Capital Medical University, Baoding 071000, Hebei, China; 5 Xin-Hui Liu Baoding Pediatric Severe Infectious Diseases Research Laboratory, Baoding Hospital of Beijing Children’s Hospital, Capital Medical University, Baoding 071000, Hebei, China; 6 Jiang-tao Ma Department of Urology Surgery, Baoding Hospital, Beijing Children’s Hospital Affiliated to Capital Medical University, Baoding 071000, Hebei, China

**Keywords:** D-dimer, Diagnosis, Fibrinogen, Pediatric, Procalcitonin, Sepsis

## Abstract

**Objective:**

To evaluate the clinical value of combined detection of procalcitonin (PCT), D-dimer (DD) and fibrinogen (FIB) in the evaluation of children with sepsis.

**Method:**

This was a retrospective study. Eighty children with sepsis hospitalized in Baoding Hospital, Beijing Children’s Hospital Affiliated to Capital Medical University from January 2022 to January 2024 were selected as the experimental group, 40 non-sepsis infected children were selected and included in the control group. Ten milliliter of elbow vein blood was collected from all children after admission, and relevant tests were completed within one hour. The levels of PCT, DD and FIB were analyzed and compared between the two groups.

**Results:**

The levels of PCT, FIB, and DD were significantly increased in children with sepsis compared with those in the common infection group, and the levels of these biomarkers were significantly increased in the severe sepsis group compared with those in the mild sepsis group, with statistical significances; the favorable diagnostic efficacy of PCT alone for sepsis. The AUC of combined detection of the three indicators were higher than those of PCT, DD, and FIB alone; PCT was significantly positively correlated with CRP, DD, and FIB, with a slightly weaker correlation with WBC.

**Conclusion:**

PCT, DD and FIB may be helpful for the diagnosis and assessment of sepsis, and PCT alone has a favorable diagnostic efficacy for sepsis and is correlated with other inflammatory factors. Combined detection of PCT, DD and FIB may be considered in the diagnosis and prognosis evaluation of pediatric sepsis in clinical practice.

## INTRODUCTION

Sepsis is a systemic inflammation syndrome caused by infection, during which endotoxins produced by pathogens, exotoxins and various inflammatory mediators mediated by these toxins can induce damage to the body.^1^ Pediatric sepsis is a common type of this disease.^2^,^3^ The pathogenesis of sepsis is complex, and primarily related to infection, immunity, pathophysiological changes and inflammation of the body. Clinical studies have found that the early symptoms of sepsis in children are not typical^4^, making early diagnosis of this disease difficult. Diagnosis and treatment of this disease in children are challenged, and the functions of multiple organs and systems of the body are adversely impaired in affected children.^5^ If children with sepsis are not promptly treated, their condition will progress and evolve into septic shock or multiple organ dysfunction, which seriously threatens the life and safety of these children.

Relevant studies have confirmed that the mortality of severe sepsis in children can be up to 69%.^6^,^7^ Therefore, timely diagnosis and treatment for children with sepsis should be provided, and early diagnosis and timely medical intervention or early medical intervention are the keys to improving the prognosis of sepsis in clinical practice, with the intensive studies on the clinical value of blood indicators in the diagnosis of diseases, increasing numbers of researchers recommend using blood biomarkers in the early diagnosis of sepsis. With the accumulative application of related biological molecular theories in clinical diagnosis, it is believed that changes in some indicators may help doctors to determine the severity and changes of the disease, and indicators such as PCT and DD are of great value in the early diagnosis of sepsis.^8^ In the present study, the levels of PCT, DD and FIB were monitored in children with sepsis, and evaluated the clinical value of combined detection of PCT, DD and FIB in the evaluation of children with sepsis.

## METHODS

This was a retrospective study. Eighty children with sepsis hospitalized in Baoding Hospital, Beijing Children’s Hospital Affiliated to Capital Medical University January 2022 to January 2024 were selected as the experimental group, and divided into the sepsis group (the mild sepsis group) and the severe sepsis and septic shock group (the severe sepsis group) based on the severity of the disease. At the same time, 40 non-sepsis-infected children were selected as the control group. The general data of the three groups were comparable with no statistically significant differences(Table-I).

**Table-I:** Comparative analysis of the general data of the three groups of children (*χ̅*±*S*).

Items	The mild sepsis group	The severe sepsis group	The control group	F/χ^2^	p
n	43	37	40		
M (n, %)	22(51%)	16(43%)	22(55%)	2.88	0.09
Age (years)	8.05±2.14	8.13±2.30	8.33±2.27	0.67	0.50
Onset time (hours)	13.04±5.87	12.63±4.93	13.87±5.26	1.33	0.19
BMI (kg/m^2^)	20.13±1.52	20.35±1.32	20.28±1.10	0.62	0.54
Body temperature (°C)	38.76±0.24	38.83±0.31	38.68±0.17	0.28	0.81
Heart rate (/min)	110.57±3.04	114.46±3.67	108.82±2.60	0.38	0.62
Mean arterial pressure (mmHg)	80.70±1.27	80.32±1.33	79.69±1.25	0.73	0.31

p>0.05

### Ethical approval:

This study protocol was approved by the Ethics Committee of the Baoding Hospital, Beijing Children’s Hospital Affiliated to Capital Medical University (No.: 202338; Date: June 12, 2023), and all guardian of the participants signed the informed consent.

### Inclusion criteria:

- Who met the clinical diagnostic criteria for sepsis.
- With an age of 5-14 years old.
- With no cognitive or mental disorders, and able to understand and actively cooperate with treatment plans.
- Whose family members agreed to participate in the study and signed informed consent forms;
- With complete clinical data.
- Who was able to cooperate in the study, with good treatment compliance.

### Exclusion criteria:

- With a time from onset to hospital visit of more than three days.
- With severe mental disorders or cognitive impairments.
- With poor treatment compliance and inability to cooperate with treatment.
- Who received glucocorticoid treatment within one month prior to admission.
- Who were unable to cooperate in the study.
- complicated with hematological diseases and malignant tumors.

After admission, 10 ml of elbow vein blood was collected from all patients, and the samples were routinely separated by high-speed centrifugation. Relevant tests were carried out within one hour after serum collection. PCT was determined using MAGLU-M11000 automatic chemiluminescence analyzer and related reagents with electrochemiluminescence method; and DD and FIB were detected using Hitachi 7600 automatic biochemical analyzer and related reagents (Hitachi, Japan) with immunoturbidimetry method. The measurement of PCT, DD, and FIB were conducted strictly according to the instructions provided with the corresponding kits. All patients in this study were routinely examined for blood analysis, urine analysis and chest radiographs. For patients with abnormal body temperature, examinations such as erythrocyte sedimentation rate and chest CT were added.

### Outcome measures:

The levels of PCT, DD, and FIB were analyzed and compared among the three groups of pediatric patients; and the specificity, accuracy and sensitivity of the combined detection of blood PCT, DD, and FIB versus those of each individual indicator in the diagnosis of sepsis were analyzed and compared.

### Statistical analysis:

Data were statistically analyzed using SPSS 20.0 software. Measurement data were presented as (*χ̅*±*S*), and enumeration data were presented as absolute values or composition ratios. Analysis of variance was used for comparison between groups, and rates were compared using c^2^ test. Influencing factors were analyzed using the logistic regression analysis method. The area under the curve (AUC) and optimal diagnostic cutoff values of PCT, DD, FIB, and combined detection were analyzed. Sensitivity, specificity, and misdiagnosis rates were calculated. The correlation between PCT and various inflammatory indicators was presented as the Pearson correlation coefficient. Differences with a p-value of <0.05 were considered statistically significant.

## RESULTS

The levels of PCT, FIB, and DD were significantly increased in children with sepsis compared with those in the common infection group, and the levels of these indicators were significantly increased in the severe sepsis group compared with those in the mild sepsis group, with statistical significances (p=0.00) (Table-II).

**Table-II:** Differences in levels of PCT, DD, and FIB among the three groups of children(*χ̅*±*S*).

Indicators	The severe sepsis group	The mild sepsis group	The control group	F	p
n	37	43	40		
PCT(ng/L)	5.06±1.24	3.70±1.18	1.16±0.42	4.27	0.00
DD(mg/L)	7.08±2.35	4.67±1.83	0.48±0.05	6.53	0.00
FIB(g/L)	7.62±1.07	5.10±1.21	3.47±1.06	6.72	0.00

p<0.05

Multivariate logistic regression analysis was performed using PCT, DD, and FIB as independent variables, with alpha to enter =0.05 and alpha to remove =0.10, and the results indicated that PCT, DD, and FIB were related indicators for sepsis(Table-III).

**Table-III:** Multivariate logistic regression analysis of PCT, DD, and FIB in children with sepsis.

Variables	Assignment	β	SE	Wald χ2	P	OR	95.0%CI
PCT*	None=0, S=1	2.532	0.756	5.129	0.000	4.552	3.526-5.810
DD	None=0, S=1	3.061	0.582	3.244	0.000	3.205	2.618-4.412
FIB*	None=0, S=1	3.028	0.408	4.385	0.000	3.237	2.509-4.233

* p<0.05 Note: S= sepsis.

Diagnostic value analysis of PCT, DD, FIB, and the combined detection for sepsis (Table-IV) showed that the AUC of PCT was 86.3%, with a sensitivity of 83.230%, and a specificity of 86.417%, which were higher than those of DD and FIB, indicating the favorable diagnostic efficacy of PCT alone for sepsis. The AUC of combined detection of the three indicators was 93.0%, with a specificity of 94.612%, and a sensitivity of 87.506%, which were higher than those of PCT, DD, and FIB alone, indicating the best diagnostic efficacy of the combined detection of the three indicators. PCT was significantly positively correlated with CRP, DD, and FIB (P=0.00), with a slightly weaker correlation with WBC(p=0.03). There was no significant correlation between PCT and ESR(p=0.433) (Table-V).

**Table-IV:** The diagnostic value of PCT, DD, FIB, and the combination of these indicators for sepsis.

Indicators	Cutoff value	Sensitivity%	Specificity%	Missed diagnosis rate%	Misdiagnosis rate %	AUC	95.0%CI
PCT (ng/L)	3.764	83.230	86.417	16.770	13.583	0.863	0.760-0.891
DD(mg/L)	6.538	82.509	84.528	17.491	15.472	0.831	0.796-0.854
FIB(g/L)	5.814	80.385	83.716	19.615	16.284	0.817	0.772-0.849
Combined detection	7.440	87.506	94.612	12.494	5.388	0.930	0.904-0.983

**Table-V:** Correlation analysis of PCT with other inflammatory factors (*χ̅*±*S*)(n=80).

Statistics	WBC(×10^9^)	CRP(mg/L)	DD(mg/L)	ESR(mm/h)	FIB(g/L)
r	0.018	0.388	0.406	0.045	0.372
p	0.03	0.00	0.00	0.433	0.00

*p<0.05.

## DISCUSSION

It was confirmed in the present study that the level of PCT was positively correlated to the severity of the disease in children with sepsis(p=0.00); multivariate logistic regression analysis suggested that PCT was a related indicator for sepsis (p=0.000); the AUC of PCT was 86.3%, with a sensitivity of 83.230% and a specificity of 86.417%, which were higher than those of DD and FIB. Further analysis showed that PCT was one of the important diagnostic indicators for sepsis, and dynamic monitoring of PCT can guide the treatment of sepsis, with an increasingly significant role in the diagnosis, treatment, and prognosis evaluation of pediatric sepsis.

The accuracy of this examination is significantly better than conventional inflammatory indicators. PCT is superior to blood culture in terms of convenience in examination and dynamic observation, making it an important indicator for predicting the disease progression of pediatric sepsis in clinical practice. PCT is a precursor peptide of serum calcitonin and is composed of 116 amino acids. The level of serum PCT in healthy individuals is extremely low and almost undetectable.^9^ When severe bacterial infections, sepsis, septicemia, and multiple organ failure occur, the level of PCT starts to increase two to three hours after endotoxins and inflammatory cytokines are induced in the body by bacteria, and peaks at six hours, followed by a decline at 24 hours. The half-life of PCT is 25-30 hours, and the favorable stability in vitro makes it easy to detect. In addition, the level of PCT significantly increases as the condition worsens.^10^

However, the level of PCT does not increase or slightly increase in viral infection, trauma, or local infection. Studies have shown that PCT is of important reference value in the differential diagnosis of bacterial and non-bacterial infections, and the level of PCT is directly proportional to the severity of infection.^11^ In severe cases, multiple organ failure may occur and the mortality of the disease increases.^12^ Therefore, early diagnosis and timely treatment are the keys to improving prognosis and reducing the mortality of children with sepsis. The condition is prone to change to various degrees in children with sepsis, which brings difficulties to the treatment of this disease. If only targeted treatment is provided and key indicators are not monitored in these pediatric patients, the pertinency and effect of treatment will be adversely affected, and children will not be treated in time after the deterioration of their condition, which easily threatens the life safety of children. Some key indicators need to be selected by doctors for monitoring.

However, various types of indicators are available for monitoring in children with sepsis, and the choice of indicators that are more pertinent needs to be discussed by doctors to select efficient, accurate and highly targeted indicators to ensure the overall efficacy and prognosis of children. Blood indicators for sepsis currently used in children include routine blood tests, PCT, interleukin(IL)-6, and serum amyloid A(SAA) among others.^13^ However, the specificity of IL-6 and SAA is low. On this basis, it is of great value to discover rapid and accurate biological indicators to improve the diagnosis rate of sepsis.

D-dimer(DD) is the fragment with the smallest molecular weight among fibrin degradation products and also the characteristic degradation product of crosslinked fibrin. An increased level of DD indicates the hypercoagulable state in the body and reflects the activation of the coagulation and fibrinolysis system.^14^ Studies have shown that dysfunction of coagulation and fibrinolysis system may occur in sepsis, and the levels of serum DD and FIB in these children are abnormal^15^, suggesting that the functional status of the blood coagulation system in children with sepsis can be understood and the microcirculation thrombosis that is not easily discovered can be found by detecting the levels of serum DD and FIB, which can guide the clinicians to timely provide anticoagulation treatment to prevent the disease from further aggravation.^16^

Therefore, DD can serve as an indicator of the severity of sepsis. FIB is one of the important indexes for coagulation function, and abnormal coagulation often predicts the aggravation of sepsis.^17^ Abnormal coagulation may occur during the course of the disease development of sepsis and is also one of the key factors for the clinical prognosis of this disease.^18^ Children with severe sepsis are often complicated with disseminated intravascular coagulation (DIC). The activation of the fibrinolytic system induced by the strong stimulation of inflammatory response may lead to the activation of a large amount of fibrin, which was consistent with the findings of the present study, i.e., the levels of FIB and DD were significantly increased in children with sepsis compared with those in the common infection group, and those in the severe sepsis group were significantly increased compared with those in the mild sepsis group, with statistical significances(p=0.00). Multivariate logistic regression analysis showed that DD and FIB were both related indicators for sepsis(p=0.000).

The ROC curve analysis confirmed that the AUC of PCT was 86.3%, with a sensitivity of 83.230% and a specificity of 86.417%; the AUC of DD was 83.1%, with a sensitivity of 82.509% and a specificity of 84.528%; the AUC of FIB was 81.7%, with a sensitivity of 80.385% and a specificity of 83.716%; and the AUC of combined detection was 93.0%, with a specificity of 94.612% and a sensitivity of 87.506%, which were higher than those of PCT, DD, and FIB alone, revealing the best diagnostic efficacy of the combined detection of the three indicators. The level of DD is relatively low in normal individuals and is generally difficult to detect.

Studies have found that the increase in serum DD is closely related to the prognosis of sepsis, making it a new target for sepsis diagnosis.^19^ FIB is involved in the coagulation process and plays an important role in determining the development and progression of diseases such as thrombosis and platelet aggregation due to its positive correlation with blood viscosity.^20^ The coagulation of blood in patients with sepsis is activated to various degrees. Apoptosis of endothelial cells or other cells can lead to the exposure of phospholipid components of procoagulant factors and consequently an increase in FIB. Therefore, the sensitivity and specificity of the combined detection of these indicators in the diagnosis of sepsis significantly increase.

### Limitations:

The limitations of the present study included the small sample size. Future studies with large sample sizes and increased follow-up are needed for more accurate evaluation of the clinical diagnostic value of PCT, DD and FIB. Early diagnosis and evaluation of pediatric sepsis are needed to hopefully benefit more children.

## CONCLUSIONS

The diagnostic value of combined detection of serum PCT, DD and FIB in children with sepsis is higher than that of each individual indicator alone, and the sensitivity and specificity are up to 94.612 % and 87.506 %, respectively, with clinical significance for determining the severity of the disease. Combined detection of PCT, DD and FIB may be considered in the diagnosis and prognosis evaluation of pediatric sepsis in clinical practice.

### Authors’ Contributions:

**HD** and **XH:** Had full access to all the data in the study and took responsibility for the integrity of the data and the accuracy of the data analysis.

**LS** and **CF:** Literature search, Study concept and design.

**XL** and **JM:** Acquisition of data, analysis, and interpretation of data.

All authors gave final approval of the version to be published.
